# The SARS-CoV-2 main protease induces neurotoxic TDP-43 cleavage and aggregates

**DOI:** 10.1038/s41392-023-01386-8

**Published:** 2023-03-09

**Authors:** Jiaxin Yang, Yan Li, Shijin Wang, Huili Li, Lili Zhang, Haichen Zhang, Pei-Hui Wang, Xiangyu Zheng, Xiao-Fang Yu, Wei Wei

**Affiliations:** 1 Institute of Virology and AIDS Research, First Hospital, Jilin University, Changchun, Jilin 130021 China; 2 Department of Neurology and Neuroscience Center, First Hospital, Jilin University, Changchun, Jilin 130021 China; 3 Key Laboratory for Experimental Teratology of Ministry of Education and Advanced Medical Research Institute, Cheeloo College of Medicine, Shandong University, Jinan, China; 4 Cancer Institute (Key Laboratory of Cancer Prevention and Intervention, China National Ministry of Education), The Second Affiliated Hospital, Zhejiang University School of Medicine, Hangzhou, Zhejiang China; 5 Cancer Center, Zhejiang University, Hangzhou, Zhejiang China; 6 Key Laboratory of Organ Regeneration and Transplantation of Ministry of Education, Institute of Translational Medicine, First Hospital, Jilin University, Changchun, Jilin 130021 China

**Keywords:** Microbiology, Infectious diseases

**Dear Editor**,

Neurologic manifestations associated with many COVID-19 patients, including acute infection with severe acute respiratory syndrome coronavirus-2 (SARS-CoV-2) and long COVID, have been proven by increasing evidence.^1^ To date, most studies have focused on how SARS-CoV-2 invades the nervous system and the consequent neuropathological changes.^2,3^ In contrast, the specific mechanism by which SARS-CoV-2 infection leads to neurological disease remains unclear.

TAR DNA-binding protein (TDP-43) is a primary component of insoluble aggregates associated with several devastating nervous system disorders, including amyotrophic lateral sclerosis (ALS) and multiple forms of frontotemporal lobar degeneration (FTLD).^4^ Gene mutation, abnormal phase separation and viral infection have been identified as major causes of TDP-43 aggregation.^5,6^ In this study, we investigated the effects of SARS-CoV-2 translated products on host TDP-43 and its functions.

We initially performed unbiased screening of the effects of 26 proteins encoded by the SARS-CoV-2 genome on the expression of TDP-43 (Supplementary Fig. 1a) and noted a specific band of approximately 36 kDa in the immunoblotting data for TDP-43 only in the presence of the main viral protease Nsp5/Mpro/3CLpro. Other examined viral proteins, including SARS-CoV-2 papain-like protease Nsp3, had no detectable influence on the expression levels of TDP-43 (Fig. 1a and Supplementary Fig. 1b). By detecting endogenous TDP-43, we subsequently confirmed that the novel band was an Nsp5-cleaved form of TDP-43 (Fig. 1b). By using the N-terminal-tagged TDP-43 ectopic expression system, the detected band was found to be an N-terminal TDP-43 product of viral Nsp5 cleavage, termed TDP-43 NTF (Supplementary Fig. 1c).

**Fig. 1:**
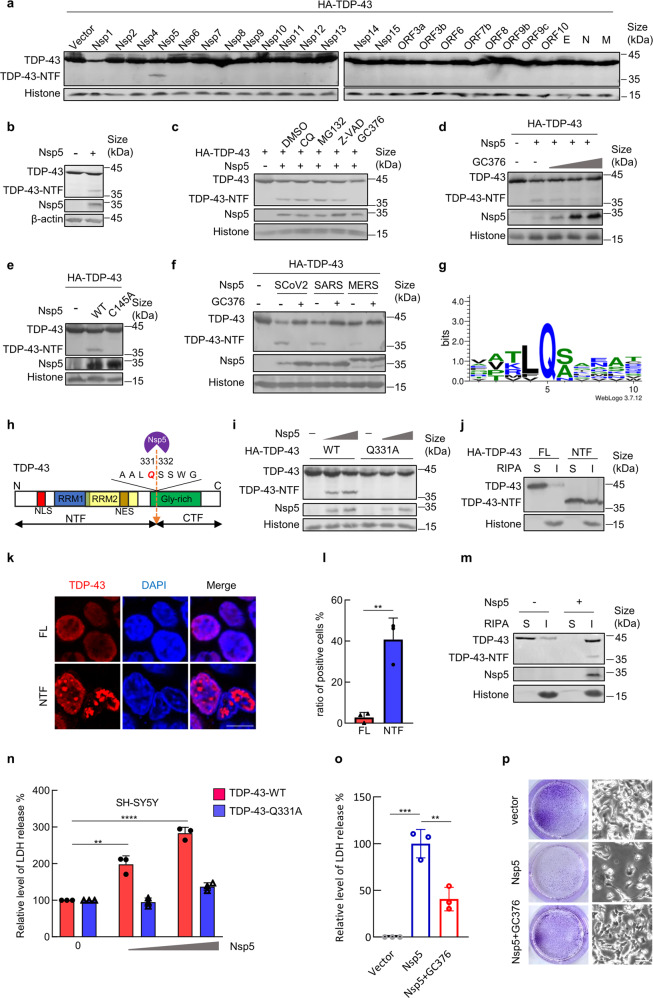
The main SARS-CoV-2 protease Nsp5 cleaves the TDP-43 protein into a cytotoxic form in human neural cells. **a** Expression plasmids of SARS-CoV-2-encoded proteins were cotransfected with pVR1012-HA-TDP-43 into HEK293T cells. Forty-eight hours later, samples were prepared for immunoblotting by using an anti-HA antibody. FL full length, NTF N-terminal fragment. **b** Immunoblotting data of endogenous TDP-43 cleaved by Nsp5. **c** Transfected HEK293T cells from (**b**) were treated with the indicated inhibitors. TDP-43 cleavage was detected by immunoblotting. CQ, chloroquine; Z-VAD, Z-VAD-FMK. DMSO was used in the solvent control group. **d** TDP-43 cleavage was inhibited by treatment with increasing concentrations of GC376 (0, 1, 5 or 20 μM). **e** HEK293T cells were cotransfected with pVR1012-HA-TDP-43 and pCAG-SARS-CoV-2-Nsp5-FLAG or its mutant Q145A. Samples were prepared for immunoblotting 48 h later. **f** Immunoblotting for TDP-43 cleaved by Nsp5 from SARS-CoV-2, SARS-CoV, and MERS-CoV. Transfected cells were treated with DMSO and GC376. **g** Logo analysis of the predicted cleavage site of SARS-CoV-2 Nsp5 by WebLogo3.7.12. **h** Schematic diagram of SARS-CoV-2-Nsp5 cleavage of TDP-43. NLS nuclear localization signal, NES nuclear export signal, RRM RNA recognition motif, Gly-rich glycine-rich domain. **i** The TDP-43 Q331A mutant is resistant to SARS-CoV-2 Nsp5. HEK293T cells were cotransfected with pVR1012-HA-TDP-43 or Q331A with increased expression vectors of SARS-CoV-2-Nsp5 (0, 100, and 200 nM). Cells were harvested at 48 h after transfection for immunoblotting assays. **j** Immunoblotting assays of the solubility of TDP-43 wild-type and NTF proteins. HEK293T cells were transfected as indicated and then collected after 48 h. Proteins were sequentially extracted using RIPA and 7 M urea buffers. S, RIPA soluble fraction; I, RIPA insoluble fraction. **k** Subcellular location of the indicated TDP-43 proteins. Scale bar, 10 μm. **l** The percentage of TDP-43 aggregates in cultures from (**k**). **m** Nsp5 decreases TDP-43 solubility. **n** SARS-CoV-2 Nsp5 enhances TDP-43 toxicity to SH-SY5Y human neuroblastoma cells. The release of LDH into the medium was used as an indicator of cytotoxicity. LDH levels were measured 72 h after cells were transfected with the indicated constructs. **o** GC376 relieved the cytotoxic effects of Nsp5 in human neuroblastoma cells. **p** Crystal violet staining (left panel) and bright-field (right panel) images of SH-SY5Y cells transfected with SARS-CoV-2 Nsp5 and treated with DMSO or 30 μM GC376. Scale bar, 50 μM. Error bars denote SEM; ANOVA test, *n* = 3 biologically independent experiments; ****p < 0.0001, ***p < 0.001, **p < 0.01

Mutant TDP-43 proteins are frequently cleaved by intracellular caspases in neurodegenerative diseases.^5^ To explore the mechanism by which viral Nsp5 cleaves TDP-43, several inhibitors were used, including a lysosomal inhibitor (chloroquine, CQ), proteasome inhibitor (MG132), pancaspase inhibitor (Z-VAD), and viral 3CLpro inhibitor (GC376).^7^ Only GC376 treatment blocked TDP-43 cleavage by Nsp5, while the others showed no significant effect (Fig. 1c). Previous studies, however, have reported the inhibitory effects of MG132 and Z-VAD-FMK against the enzyme activity of Nsp5 using a fluorescence resonance energy transfer (FRET)-based assay in vitro.^8^ The discrepancy may be due to the usage of the different evaluation systems. Moreover, inhibition of GC376 occurred in a dose-dependent manner (EC50 = 5 μm) without cytotoxicity (Fig. 1d and Supplementary Fig. 2), and TDP-43 cleavage was abolished in the activity-deficient Nsp5-C145A mutant (Fig. 1e). Overall, SARS-CoV-2 shows distinct strategies, from TDP-43 proteinopathy to direct cleavage of TDP-43 through the protease activity of Nsp5.

Considering the high degree of homology between Nsp5 proteins from SARS-CoV-2 and other coronaviruses, including severe acute respiratory syndrome coronavirus (SARS-CoV) and Middle East respiratory syndrome coronavirus (MERS-CoV) (Supplementary Fig. 3), we further determined that all three tested Nsp5 proteins are able to cleave TDP-43 (Fig. 1f). GC376 treatment broadly inhibited Nsp5 function. Interestingly, SARS-CoV-2 Nsp5 showed lower expression levels but higher efficiency in TDP-43 cleavage than Nsp5 from SARS-CoV or MERS-CoV. Moreover, viral protease Nsp5 from SARS-CoV-2 variants of concern (Beta and Omicron) also efficiently triggered TDP-43 cleavage (Supplementary Fig. 4). These results suggest that TDP-43 is a common substrate for coronavirus Nsp5.

Multiple sequence alignment using MEGA and logo analysis of identified SARS-CoV-2 Nsp5 cleavage sites revealed that this viral protease preferentially recognizes substrates containing the core sequence “LQS” for proteolytic cleavage (Fig. 1g). According to the molecular size of the cleavage product we detected, we found similar motif sequences centered at 331Gln (Fig. 1h). Furthermore, the mutant TDP-43-Q331A was resistant to cleavage by coronavirus Nsp5 compared to wild-type TDP-43 (Fig. 1i and Supplementary Fig. 5). Hence, SARS-CoV-2 Nsp5 cleaves TDP-43 at residue Q331.

Next, we investigated the influence of Nsp5 on TDP-43 solubility, which has been considered to be a major cause of neurotoxicity in degenerative diseases. We used a well-established TDP-43 solubility assay and confirmed that TDP-43 NTF exhibited strongly decreased solubility compared to that of wild-type TDP-43 (Fig. 1j). Fluorescent confocal microscopy further detected increased inclusions by TDP-43 NTF (Fig. 1k, l). Consistent with this result, Nsp5 expression significantly decreased the solubility of TDP-43 proteins (Fig. 1m). We noted that both cleaved and full-length TDP-43 accumulated in the insoluble fraction in the presence of Nsp5 (Fig. 1m). In addition, TDP-43 NTF interacted with full-length TDP-43 (Supplementary Fig. 6). These results indicate that Nsp5-cleaved TDP-43 NTF can serve as a dominant-negative mutant of wild-type TDP-43, which may further amplify the effects of Nsp5 on TDP-43 protein solubility and function.

Our findings led us to investigate the cytotoxic effects of Nsp5 cleavage of TDP-43 in neural cells. In SH-SY5Y human neuroblastoma cells, TDP-43 expression resulted in higher cytotoxicity with increased LDH release in the presence of Nsp5 in a dose-dependent manner (Fig. 1n). We used the Nsp5-resistant mutant TDP-43-Q331A as a negative control, which had no influence on TDP-43 cytotoxicity, with or without Nsp5 expression (Fig. 1n). Similar results were also observed in T98G human glioblastoma cells (Supplementary Fig. 7). We also found that ectopic expression of TDP-43 Q331A relieved Nsp5-triggered neurotoxicity (Supplementary Fig. 8), further supporting the ability of Nsp5 to cause neural damage Nsp5 via cleavage of TDP-43.

The observed dependence of 3CLpro activity on Nsp5-mediated TDP-43 cleavage led us to measure the effects of the small-molecule inhibitor GC376 on Nsp5 neurotoxicity. As expected, GC376 alleviated the inhibition of LDH release and neural cell proliferation triggered by Nsp5 expression without cytotoxicity (Fig. 1o, p and Supplementary Fig. 9). Thus, Nsp5 is a viral neurotoxic factor, and its 3CLpro activity is a druggable target for relieving neural damage.

SARS-CoV-2 infection results in multilineage neural cell dysregulation, decreased new neuron generation, and a reduction in overall brain size in patients, even those with mild respiratory COVID-19.^2^ The infectivity of SARS-CoV-2 in neural cells has been confirmed in human neural progenitor cells, brain organoids and nonhuman primates.^3,9,10^ Overall, there is a critical need to determine which viral factors contribute to central nervous system (CNS) disorders. Hence, our identification of Nsp5 as cytotoxic towards neuroblasts and glial cells demonstrates a direct killing effect of SARS-CoV-2 infection on neuronal cells through TDP-43 cleavage, which may lead to further CNS dysfunction, including neuroinflammation. Nevertheless, the effects of TDP-43 cleavage by the viral protease Nsp5 on host neural damage still need to be further confirmed in organoids, animal models, and infected patients.

The number of COVID-19 cases continues to rise and now exceeds 625 million worldwide, and long COVID has emerged and cannot be ignored. Considering that TDP-43 dysfunction has been well defined as a hallmark associated with several neurodegenerative diseases,^4^ including ALS, FTLD and Alzheimer’s disease, whether the devastating effect of Nsp5-mediated TDP-43 cleavage can be reversed after infection and the relationship between SARS-CoV-2 infection and a high risk of neurodegenerative-like diseases urgently need to be investigated with additional research.

## Supplementary information

Supplementary information

Original data

## Data Availability

The raw data are available from the corresponding authors.
