# Supplementary material for: The SARS-CoV-2 main protease induces neurotoxic TDP-43 cleavage and aggregates
Source: Signal Transduct Target Ther. 2023 Mar 9;8:109. doi: 10.1038/s41392-023-01386-8 (PMC9998009; doi:10.1038/s41392-023-01386-8)
Supplement: Supplementary file 1 — Supplementary information [file 41392_2023_1386_MOESM1_ESM.doc]

**Supplementary Information**

**The SARS-CoV-2 main protease induces neurotoxic TDP-43 cleavage and aggregates**

**Yang et. al**


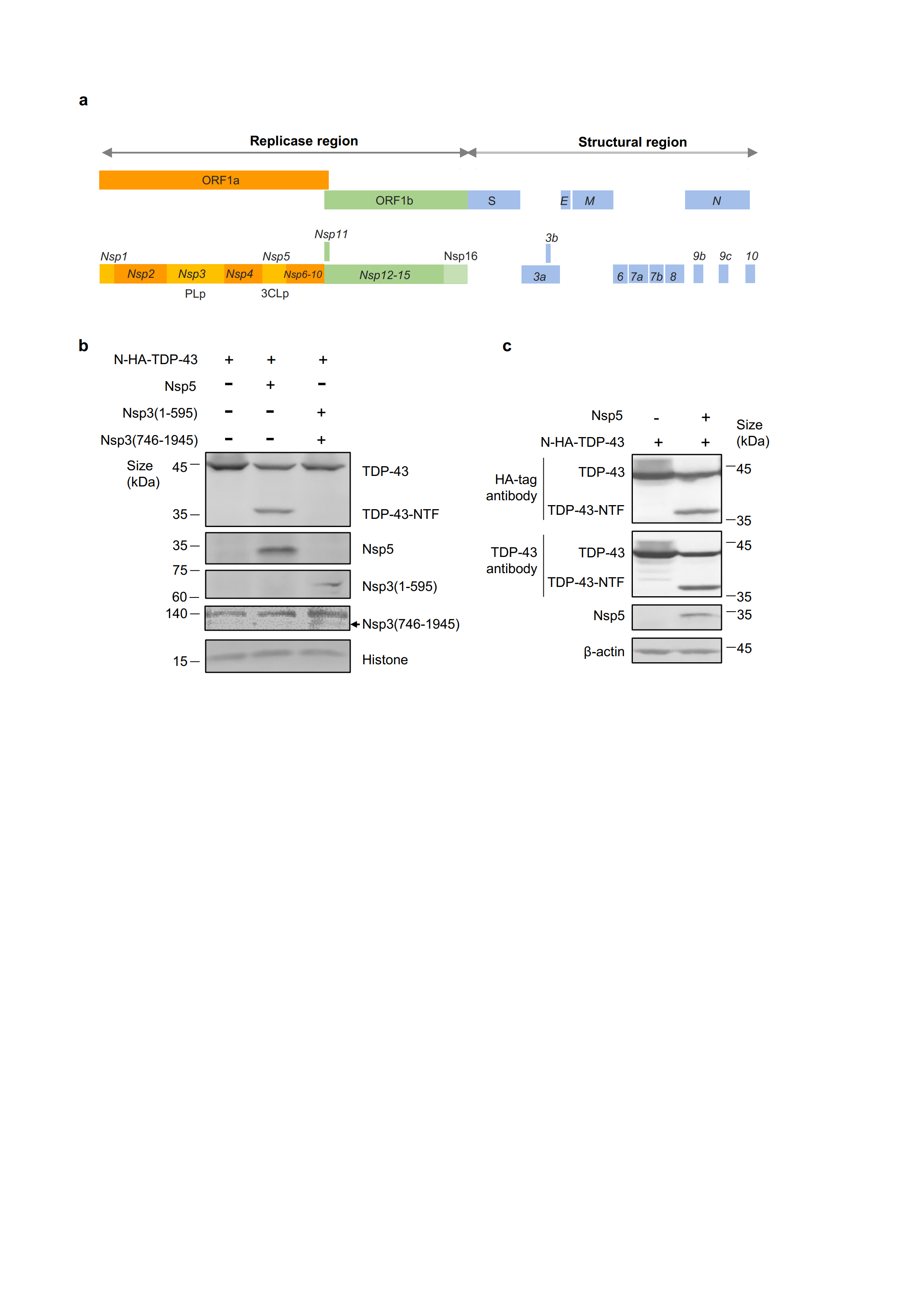


**Supplementary Figure 1.** SARS-CoV-2-Nsp5 cleaves human TDP-43 proteins. a, Diagram of the SARS-CoV-2 genome. ORF, open reading frame; S, spike; E, envelope; M, membrane; N, nucleocapsid; Nsp, nonstructural protein; PLp, papain-like protease; 3CLp, 3C-like protease. Italics indicate the proteins under investigation. b, HEK293T cells were co-transfected with pVR1012-HA-TDP-43 and pCAG-SARS-CoV-2-Nsp3(1-595)-FLAG and pCAG-SARS-CoV-2-Nsp3(746-1945)-FLAG or pCAG-SARS-CoV-2-Nsp5-FLAG; the cells were harvested after 48 h and prepared for immunoblotting using the indicated antibodies. c, pCAG-FLAG-SARS-CoV-2-Nsp5 and pVR1012-HA-TDP-43 were transfected into HEK293T cells. The transfected cells were then harvested after 48 h for immunoblotting using an anti-HA antibody or anti-TDP-43 antibody.


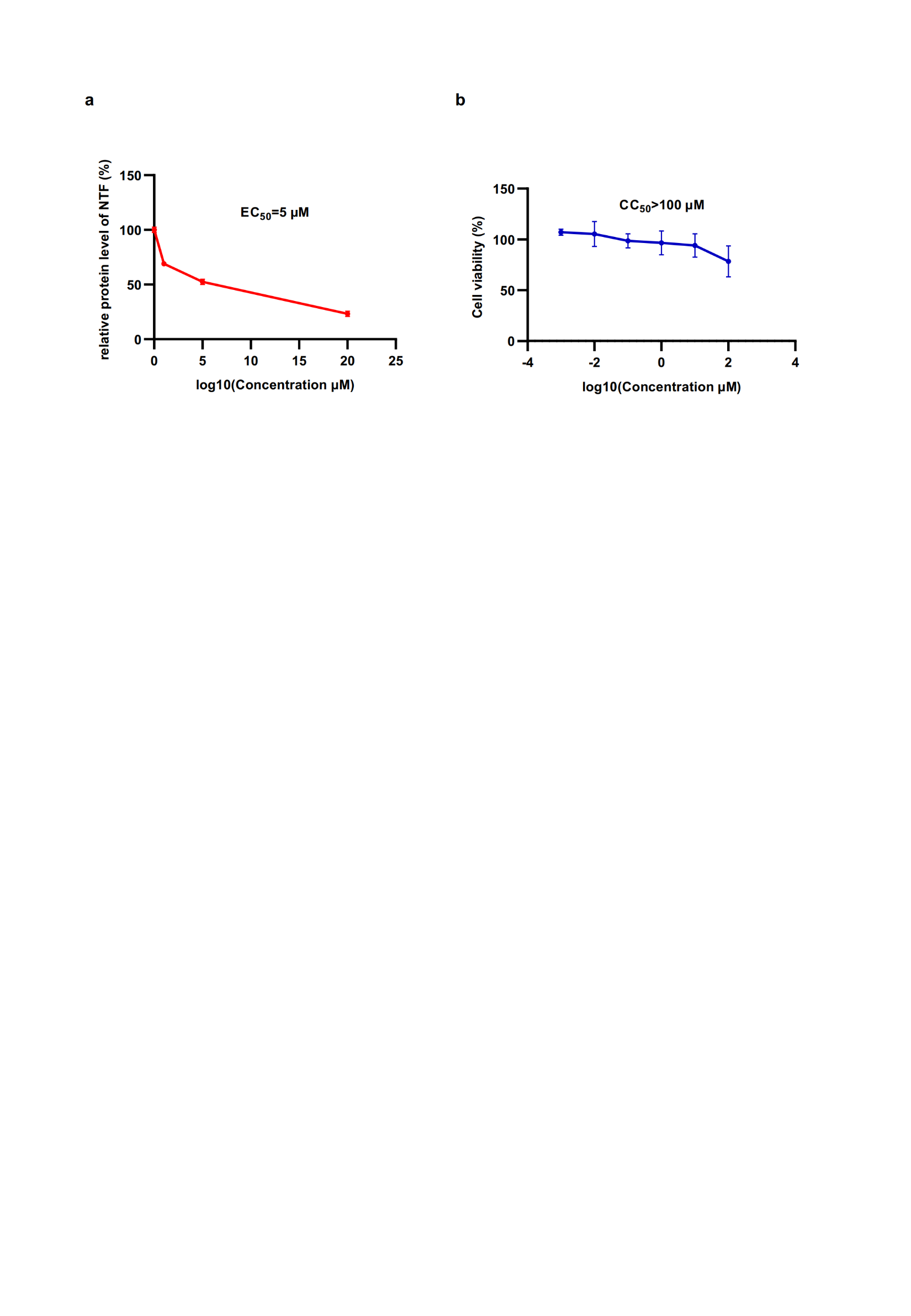


**Supplementary Figure 2. Inhibition of Nsp5-mediated TDP-43 cleavage by GC376 occurs in a dose-dependent manner without cytotoxicity.** a, EC_50_ (concentration for 50% of maximal effect) of GC376 to inhibit cleavage of TDP-43 by SARS-CoV-2-Nsp5 according to grey analysis of western blotting in Fig. 1d. b, Viability of GC376-treated cells detected using a CCK8 kit.


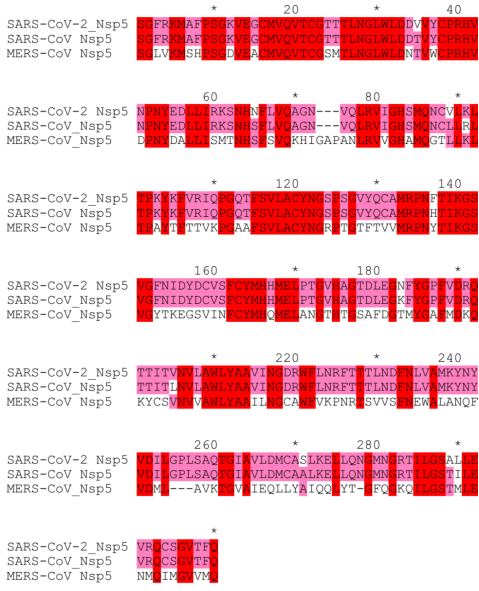


**Supplementary Figure 3.** Alignment of the amino acid sequences of Nsp5 from SARS-CoV-2 (GenBank: NC_045512.2), SARS-CoV (GenBank NC_004718.3), and MERS-CoV (GenBank NC_019843.3).


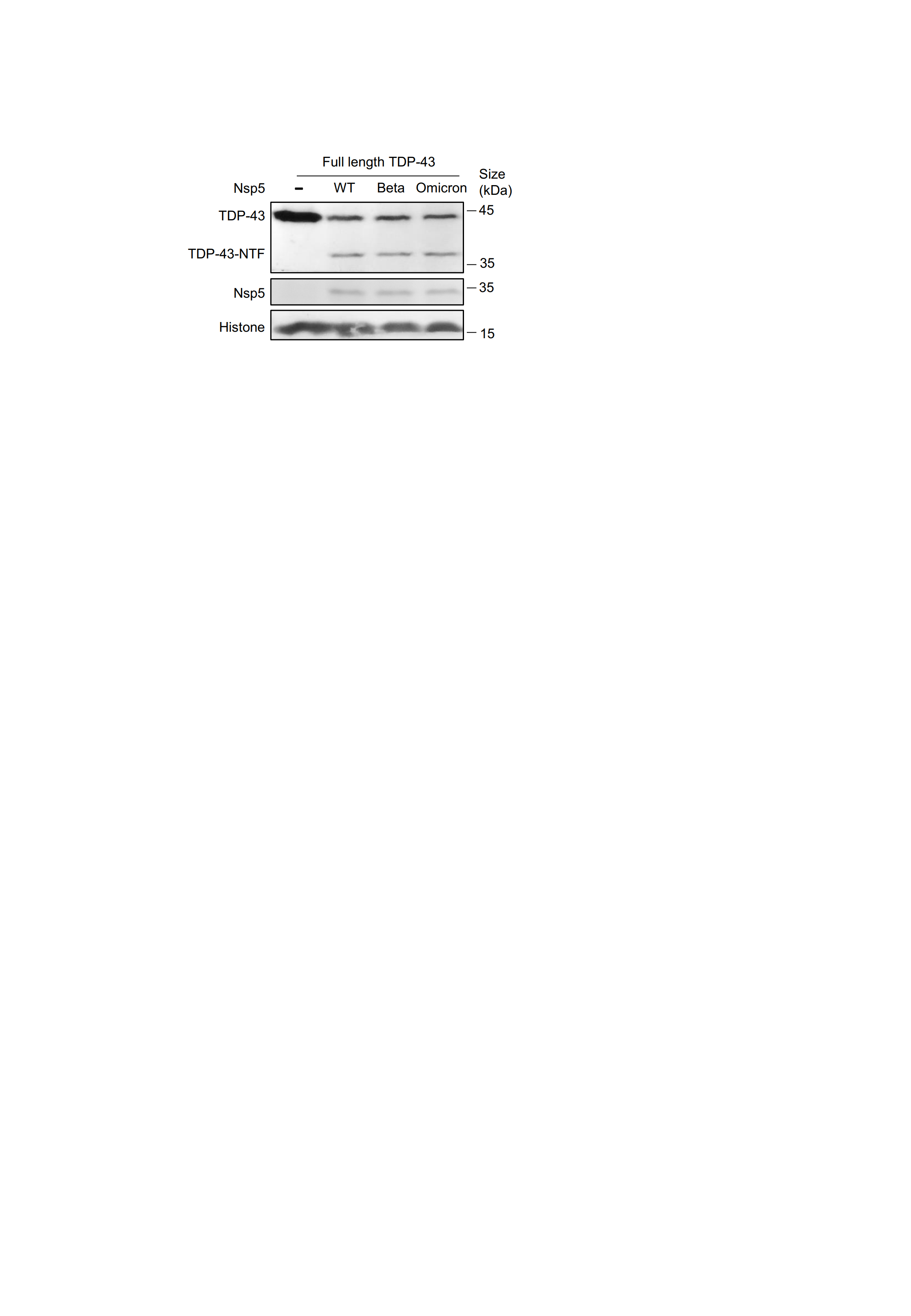


**Supplementary Figure 4.** Nsp5 from SARS-CoV-2 circulating variants induces TDP-43 cleavage. HEK293T cells were co-transfected with TDP-43 and indicated Nsp5 expression plasmids (SARS-CoV-2 wild-type Nsp5, Beta:K90R, Omicron:P132H).Cells were harvested at 48 h after transfection for immunoblotting assays.


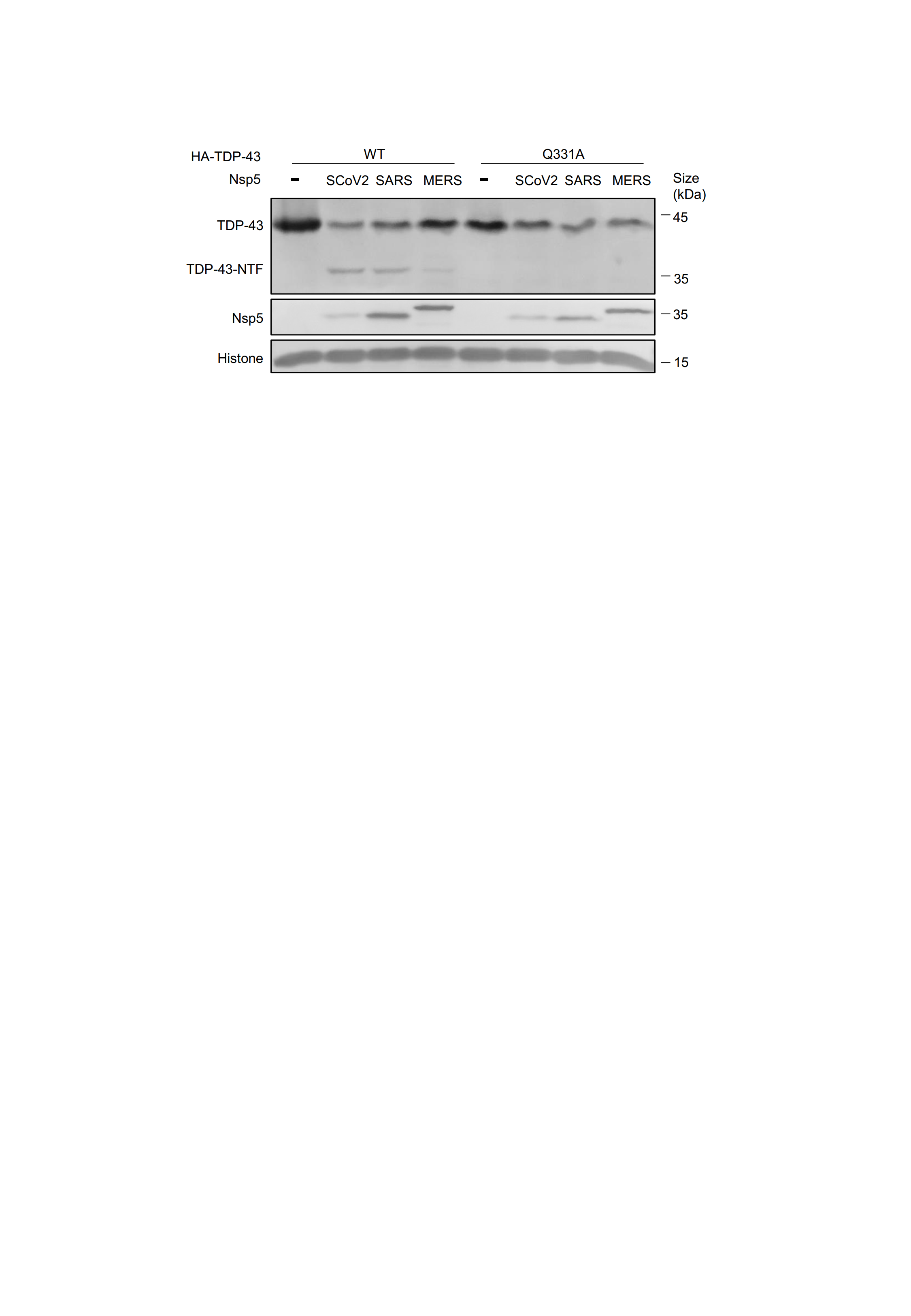


**Supplementary Figure 5.** The TDP-43 Q331A mutant is resistant to coronavirus Nsp5. HEK293T cells were co-transfected with TDP-43 wild-type or Q331A with the indicated Nsp5-expressing plasmids. Cells were harvested at 48 h after transfection for immunoblotting assays.


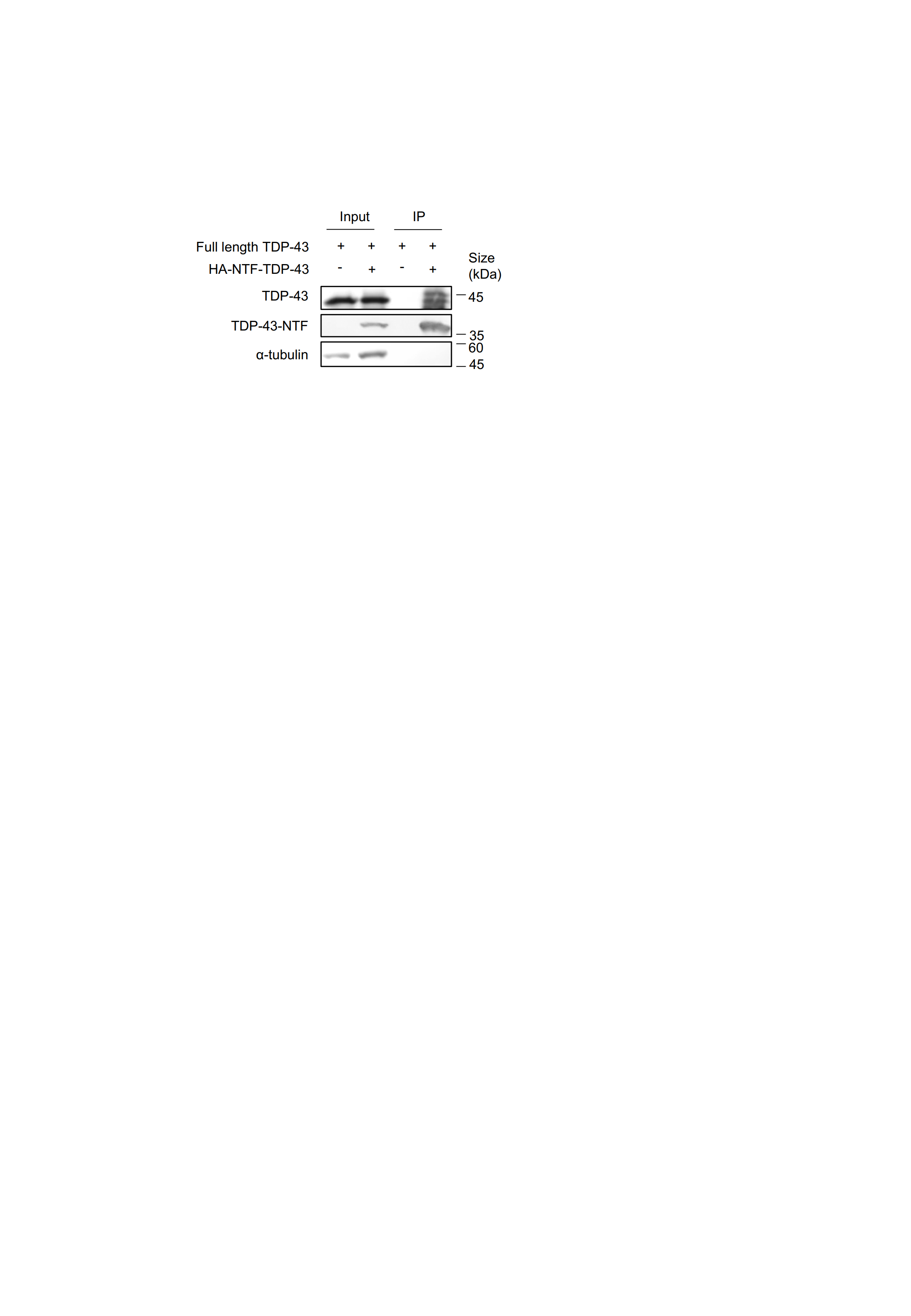


**Supplementary Figure 6.** The interaction between TDP-43-NTF and full length TDP-43. HEK293T cells were co-transfected with plasmids encoding full length TDP-43 with or without HA-TDP-43 NTF, co-immunoprecipitation experiments were performed at 48 h after transfection by using anti-HA binding beads. Samples were then subjected to immunoloblotting assays. TDP-43-NTF, TDP-43 N terminal fragment.


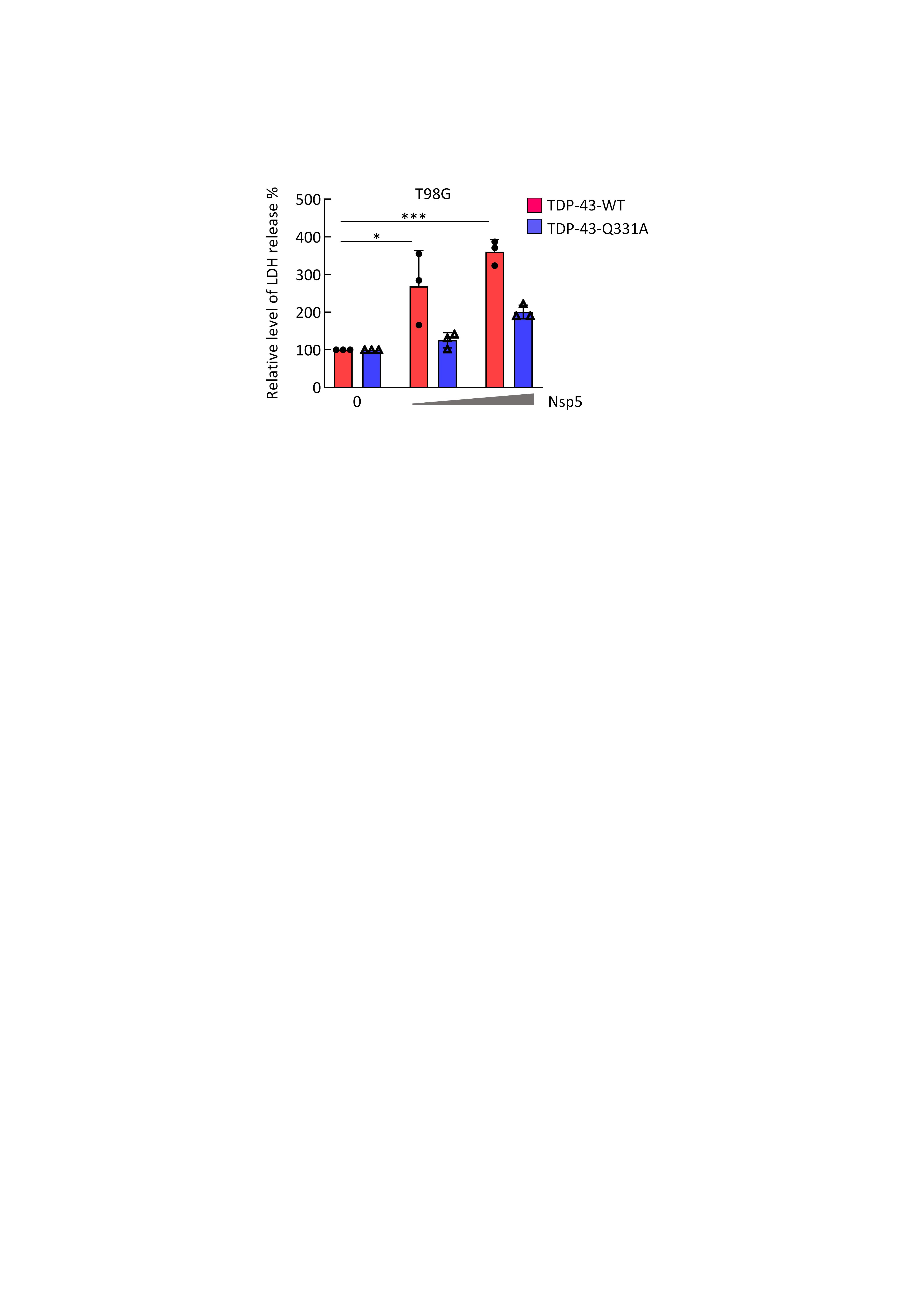


**Supplementary Figure 7.** SARS-CoV-2 Nsp5 enhances cellular toxicity in human glioblastoma cells. Release of LDH into the media was used as an indicator of cell toxicity. LDH levels were measured at 72 h after T98G cells were transfected with the indicated constructs. Error bars denote SEM; ANOVA test, n = 3 biologically independent experiments; ****p* < 0.001, **p* < 0.05.


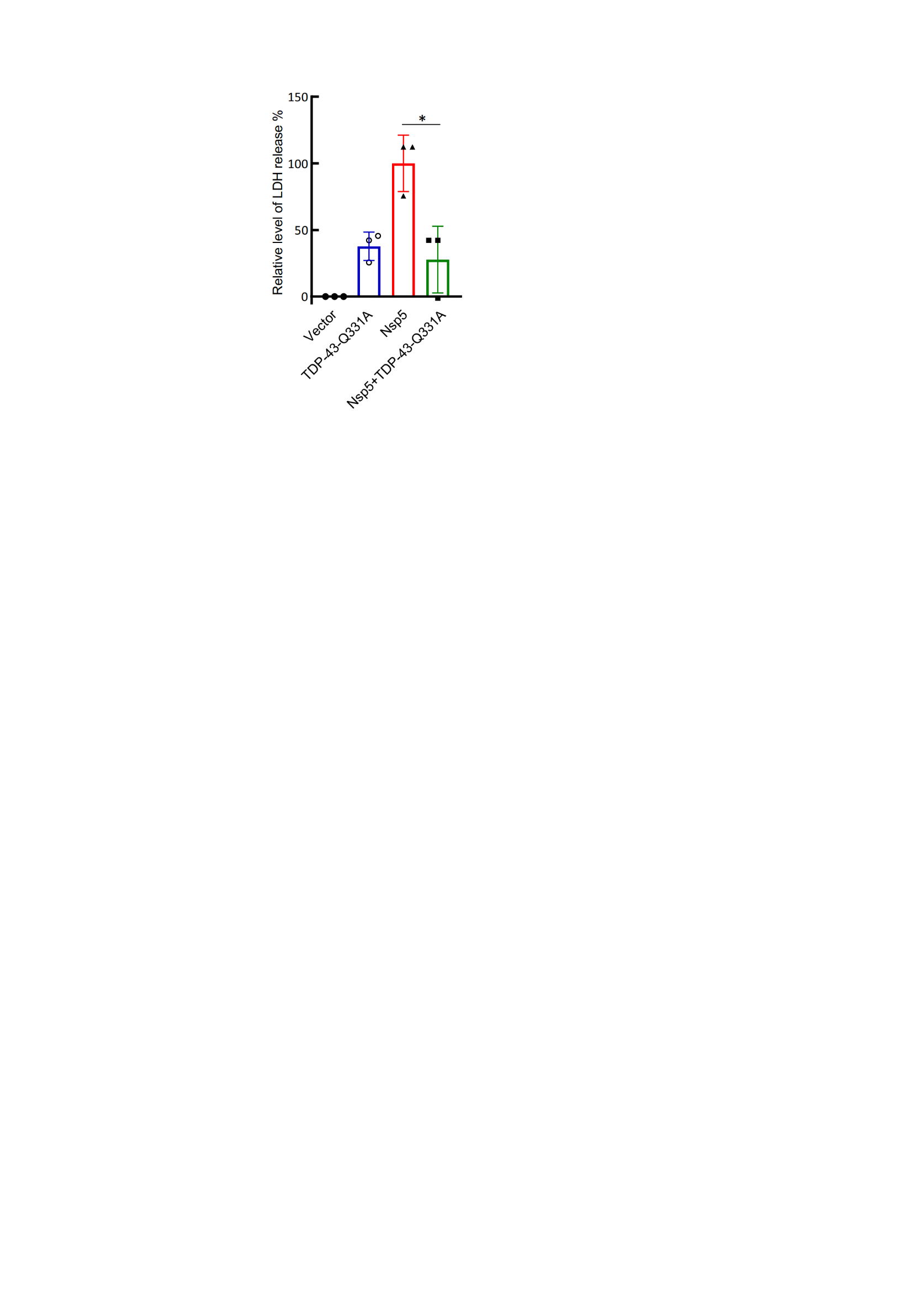


**Supplementary Figure 8.** TDP-43 Q331A relieves Nsp5-mediated LDH release in SH-SY5Y cell.


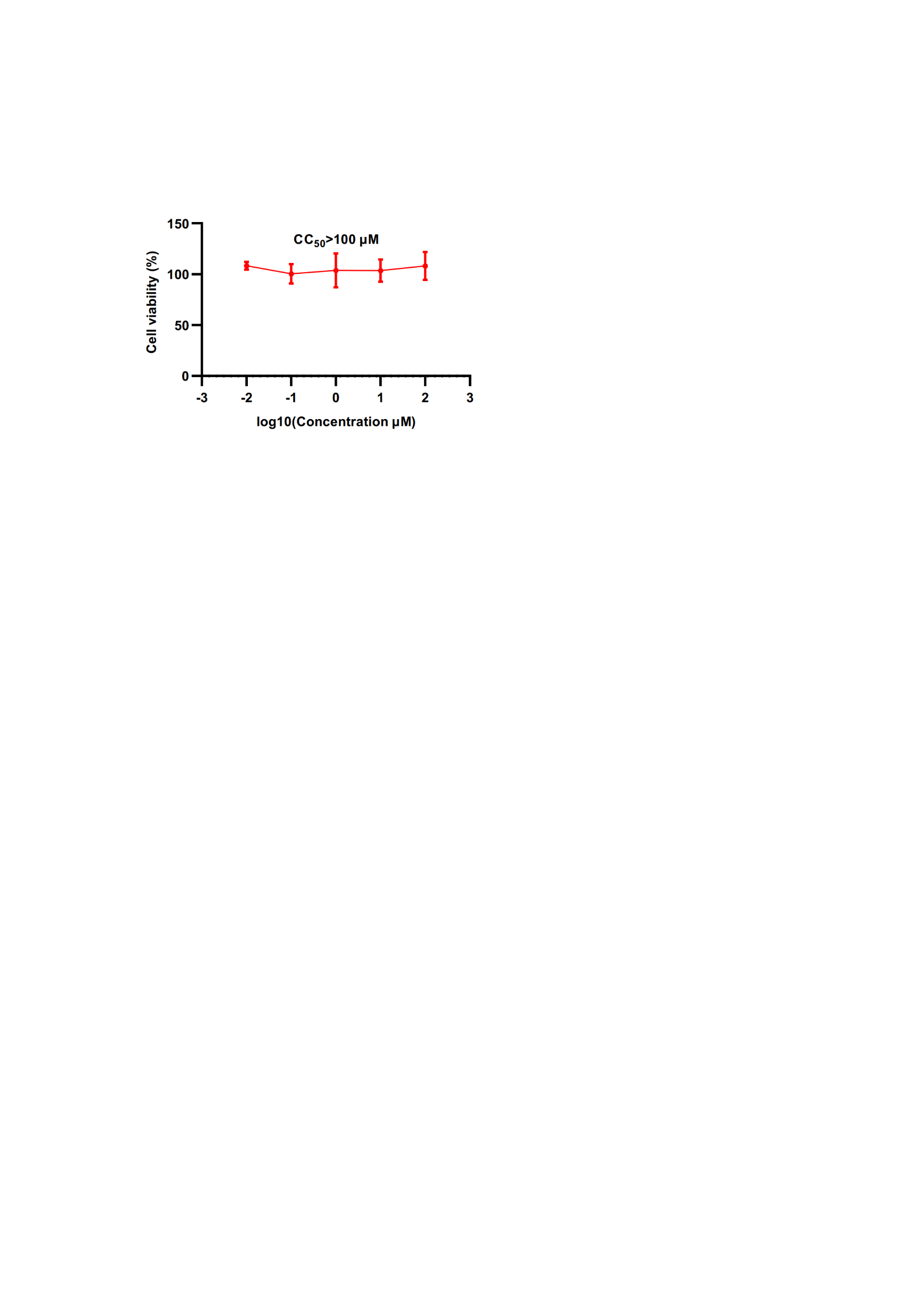


**Supplementary Figure 9.** Viability of of GC376 in SH-SY5Y cells.

**Methods**

**Plasmids and Reagents**

The plasmids pLVX-EF1alpha-SARS-CoV-2-encoding-proteins-2xStrep-IRES-Puro (Addgene 141367~141381, 141383~141394) were gifts from Dr. Nevan Krogan purchased from Addgene. pCAG-SARS-CoV-2-Nsp5-FLAG, pCAG-SARS-CoV-2-Nsp3(1-595)-FLAG and pCAG-SARS-CoV-2-Nsp3(746-1945)-FLAG were kindly provided from Dr. Pei-Hui Wang. The expression vectors pVR1012-TDP-43, pVR1012-SARS-CoV NSP5-3*FLAG and pVR1012-MERS-CoV NSP5-3*Flag were synthesized and constructed by Generay Biotech Co. Ltd (Shanghai, China). The mutant TDP-43 Q331A expressing plasmid was generated by site-specific mutagenesis from pVR1012-TDP-43.

Rabbit anti-HA antibody (71-5500) was purchased from Thermo Fisher Scientific (Waltham, MA). Mouse anti-TDP-43 antibody (ab104223) and anti-Strep-tag II antibody (ab184224) were purchased from Abcam (Cambridge, MA). Mouse anti-FLAG antibody (F1804) was purchased from Sigma-Aldrich (Milwaukee, USA). MG132 (HY-13259), Chloroquine (HY-17589A), Z-VAD-FMK (HY-16658B) were purchased from MCE (Monmouth Junction, NJ), and GC376 (S0475) was purchased from SelleckChem (Houston, TX, USA).

**Cells**

HEK293T (CRL-3216, ATCC) cells and T98G (CRL-1690, ATCC) cells were cultured in DMEM with 10% fetal bovine serum and 1% penicillin/streptomycin solution. SH-SY5Y cells (SCSP-5014, National Collection of Authenticated Cell Cultures) were cultured in MEM/F12 medium with 10% fetal bovine serum, 1% non-essential amino acids, and penicillin/streptomycin at 37°C, 5% CO_2_.

**Transfection and Immunoblotting**

DNA transfection was carried out in HEK293T and T98G cells using polyethyleneimine linear-MW40000 (24765, Polysciences, Warrington, PA). SH-SY5Y cells were transfected using Lipofectamine 2000 (Invitrogen, Carlsbad, CA, USA) according to the manufacturer's instructions. Cell samples were harvested by scraping, washed twice with cold PBS, lysed in lysis buffer (150 mM Tris, pH 7.5, with 150 mM NaCl, 1% Triton X-100, and complete protease inhibitor cocktail tablets [Roche]) and mixed with loading buffer (0.08 M Tris, pH 6.8, with 2.0% SDS, 10% glycerol, 0.1 M DTT, and 0.2% bromophenol blue).

Samples were boiled for 5 min and centrifuged at 12,000 rpm for 10 min. The cell lysates were separated via SDS-PAGE and transferred to nitrocellulose membranes (Millipore, Billerica, MA) using a semidry apparatus (Bio-Rad, Richmond, CA, USA). The membranes were probed with various primary antibodies against the proteins of interest; secondary antibodies were alkaline phosphatase-conjugated anti-goat IgG and anti-mouse IgG (Jackson ImmunoResearch Laboratories). Staining was conducted with 5-bromo-4-chloro-3-indolyl phosphate and NBT solutions prepared from chemicals obtained from Sigma-Aldrich (Milwaukee, USA). The uncropped blots are provided as a Source Data file.

**Cell Viability**

Cell viability was measured using Cell Counting Kit-8(HY-K0301, MCE) according to the instruction. Briefly, cells were cultured in a 96-well plate overnight and then treated with different concentrations of GC376. Post 48 hours, 10ul/well of CCK8 solution was added and incubated for 4 hours at 37°C. The absorbance was measured at 450nm with a microplate reader (iMark, Bio-Rad).

**Cell Fractionation**

Proteins were sequentially extracted using RIPA and urea buffers to examine the solubility of TDP-43. Briefly, cells were washed twice with PBS and lysed with cold RIPA buffer (containing complete protease inhibitor cocktail tablets and PMSF). Cell lysates were sonicated and centrifugated at 17000 rpm for 30 min at 4°C. Supernatants were RIPA-soluble fractions, while pellets were RIPA-insoluble fractions. After washing by re-sonication and re-centrifugation, RIPA-insoluble pellets were extracted with 7M urea buffer.

**Immunofluorescence**

Cells were washed twice with PBS at 48 hours post-transfected, then fixed with 4% paraformaldehyde for 30 min at room temperature, permeabilized in 0.3% Triton X-100 for 10 min, and blocked in 5% BSA solution for 1 hour. Then, cells were incubated sequentially with rabbit anti-HA antibody (71-5500, Thermo Fisher Scientific and Alexa Fluro 594 conjugated goat anti-rabbit IgG antibody (A-32740, Life Technologies). Nuclei were counterstained with 4'6-diamidino-2-phenylindole (DAPI). Fluorescence imaging was performed using a fluorescence microscope (ELWD, Nikon) with a maximum magnification of 40X.

**Toxicity Assay**

LDH release level was measured with LDH Cytotoxicity Assay Kit (40209ES76, Yeasen Biotechnology, Shanghai, CN). Briefly, the supernatants were centrifuged at 1000rpm for 10 min 72 hours post-transfection, then diluted ten-fold with PBS and incubated with LDH working solution in dark for 30 min at room temperature. The absorbance of 490/600 nm was measured by a microplate reader (iMark, Bio-Rad).

**Crystal violet Staining**

Cells in 12 well plate were washed twice with PBS and then fixed with methyl alcohol for 30 minutes at room temperature. After staining with 0.5% crystal violet solution for 1 hour, cells were rinsed with pure water and left to dry for imaging.

**Statistical Analysis**

All statistical analyses were performed using GraphPad Prism software (version 5.0). Statistical analysis was performed using a one-way ANOVA. A value of *p* < 0.05 was considered significant (**p* < 0.05; ***p* < 0.01; ****p* < 0.001).
