# Supplementary material for: The SARS-CoV-2 main protease induces neurotoxic TDP-43 cleavage and aggregates
Source: Signal Transduct Target Ther. 2023 Mar 9;8:109. doi: 10.1038/s41392-023-01386-8 (PMC9998009; doi:10.1038/s41392-023-01386-8)
Supplement: Supplementary file 2 — Original data [file 41392_2023_1386_MOESM2_ESM.pdf]

Figure 1a

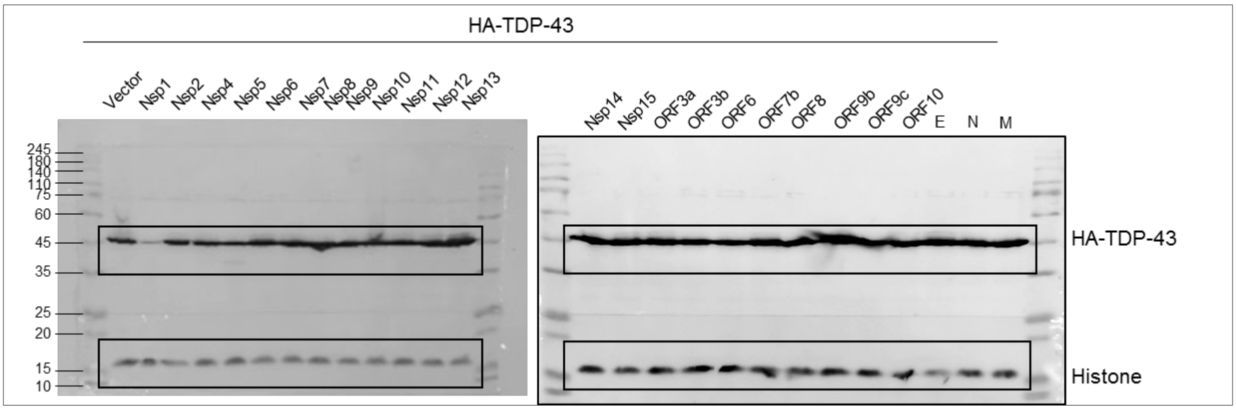
HA-TDP-43
Vector
Nsp1
Nsp2
Nsp4
Nsp5
Nsp6
Nsp7
Nsp8
Nsp9
Nsp10
Nsp11
Nsp12
Nsp13
Nsp14
Nsp15
ORF3a
ORF3b
ORF6
ORF7b
ORF8
ORF9b
ORF9c
ORF10
E
N
M
245
180
140
110
75
60
45
35
25
20
15
10
HA-TDP-43
Histone

Figure 1b

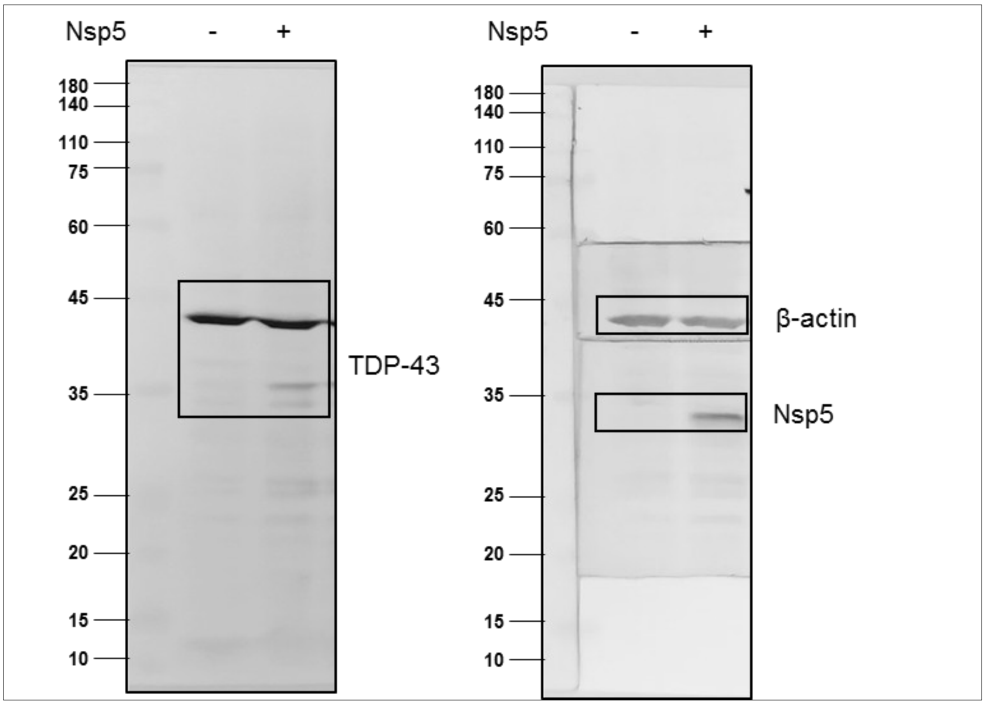
Nsp5 - +
180
140
110
75
60
45
35
25
20
15
10
TDP-43
Nsp5 - +
180
140
110
75
60
45
35
25
20
15
10
β-actin
Nsp5

Figure 1c

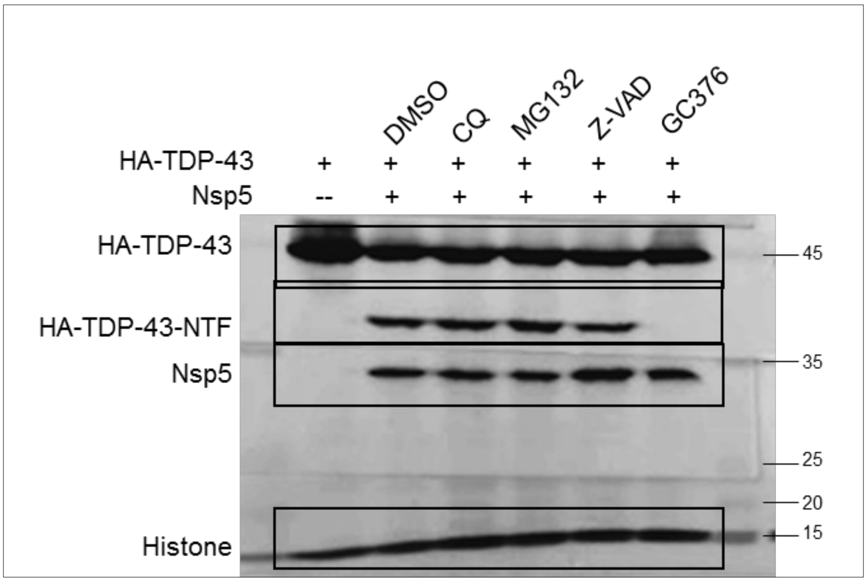
DMSO
CQ
MG132
Z-VAD
GC376
HA-TDP-43 + + + + + +
Nsp5 -- + + + + +
HA-TDP-43
HA-TDP-43-NTF
Nsp5
Histone
45
35
25
20
15

Figure 1d

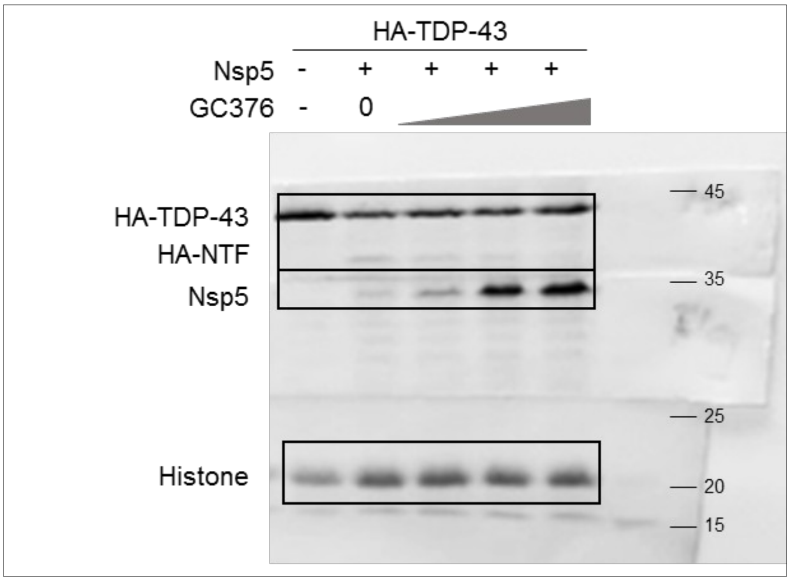
HA-TDP-43
Nsp5 - + + + +
GC376 - 0
HA-TDP-43
HA-NTF
Nsp5
Histone
45
35
25
20
15

Figure 1e

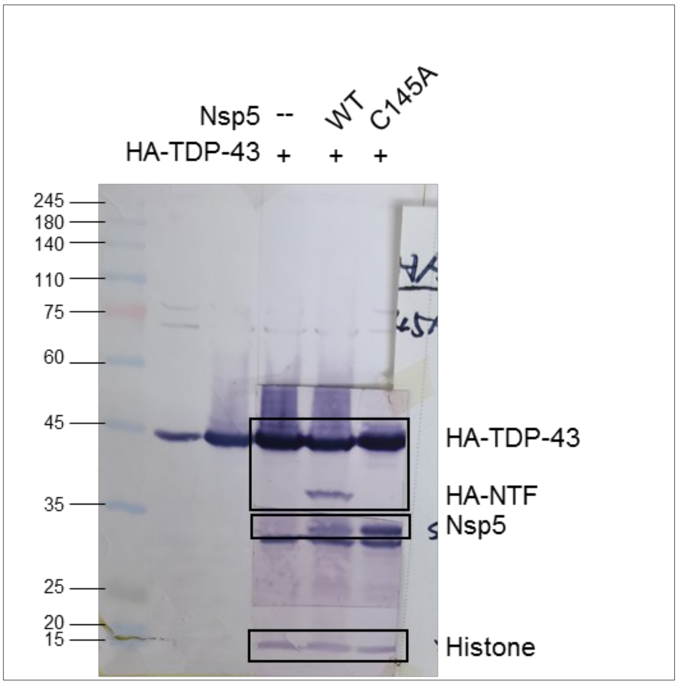
Nsp5 -- WT C145A
HA-TDP-43 + + +
245
180
140
110
75
60
45
35
25
20
15
HA-TDP-43
HA-NTF
Nsp5
Histone

Figure 1f

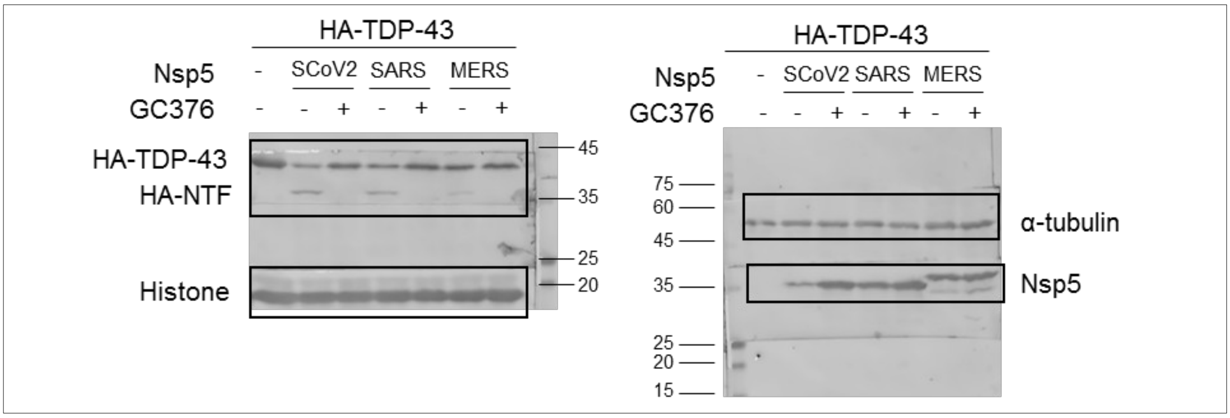

HA-TDP-43
Nsp5 - SCoV2 SARS MERS
GC376 - - + - + - +
HA-TDP-43
HA-NTF
Histone
45
35
25
20
HA-TDP-43
Nsp5 - SCoV2 SARS MERS
GC376 - - + - + - +
75
60
45
35
25
20
15
α-tubulin
Nsp5

Figure 1i

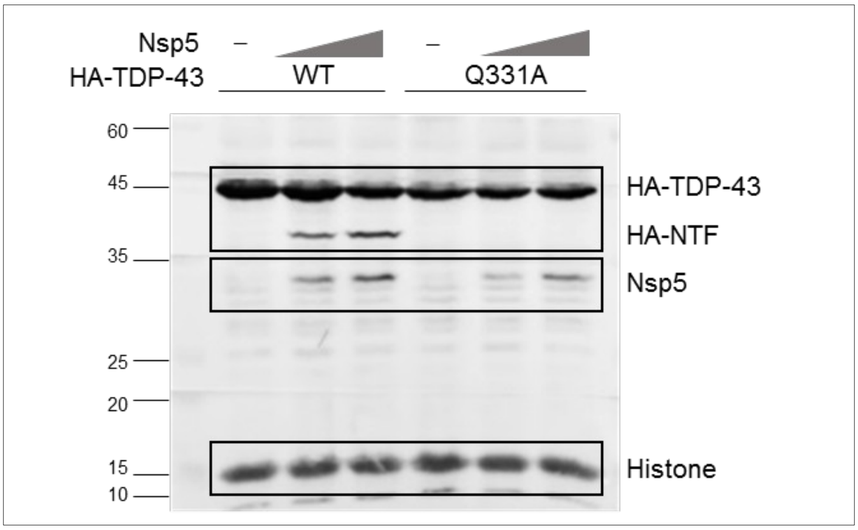
Nsp5
–
–
HA-TDP-43
WT
Q331A
60
45
35
25
20
15
10
HA-TDP-43
HA-NTF
Nsp5
Histone

Figure 1j

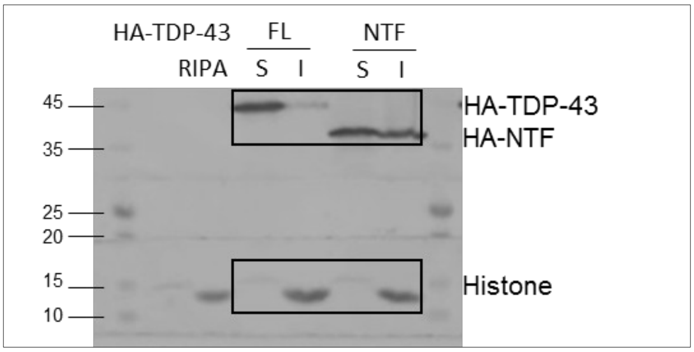
HA-TDP-43
FL
NTF
RIPA
S
I
S
I
45
35
25
20
15
10
HA-TDP-43
HA-NTF
Histone

Figure 1k

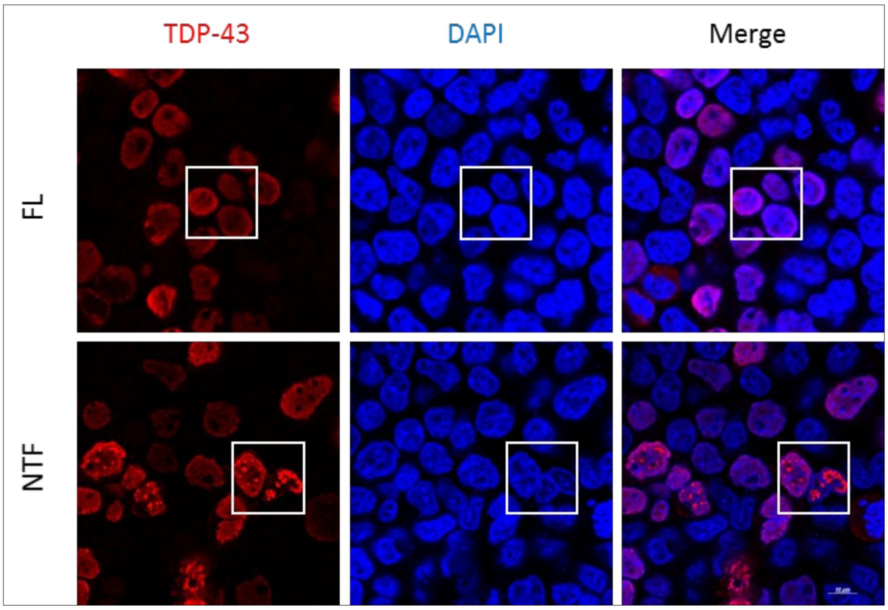

Figure 1m

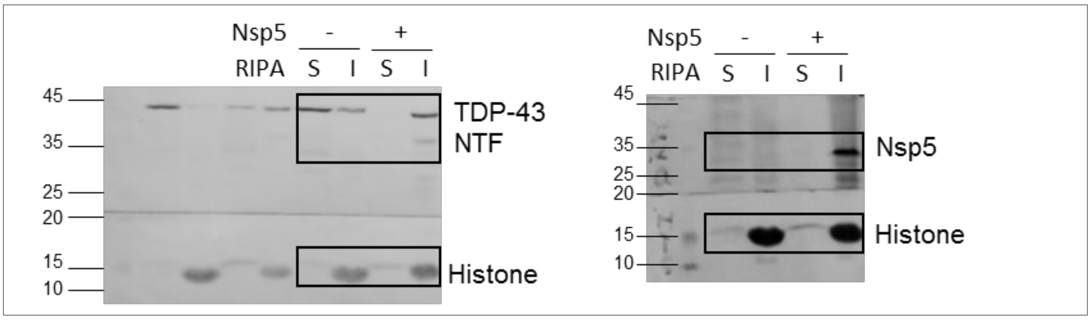

Nsp5 - +
RIPA S I S I
45
35
25
20
15
10
TDP-43
NTF
Histone
Nsp5 - +
RIPA S I S I
45
35
25
20
15
10
Nsp5
Histone

Figure 1n

SH-SY5Y

TDP-43	-	WT			Q331A		
Nsp5(ng)	0	0	100	200	0	100	200
	0.372	0.471	0.517	0.585	0.515	0.521	0.563
	0.403	0.457	0.547	0.607	0.548	0.536	0.591
	0.404	0.469	0.546	0.604	0.521	0.506	0.58

Figure 1o

SH-SY5Y

	Vector	Nsp5	Nsp5+GC376
	0.169	0.205	0.19
	0.189	0.213	0.197
	0.186	0.212	0.192

Figure 1p

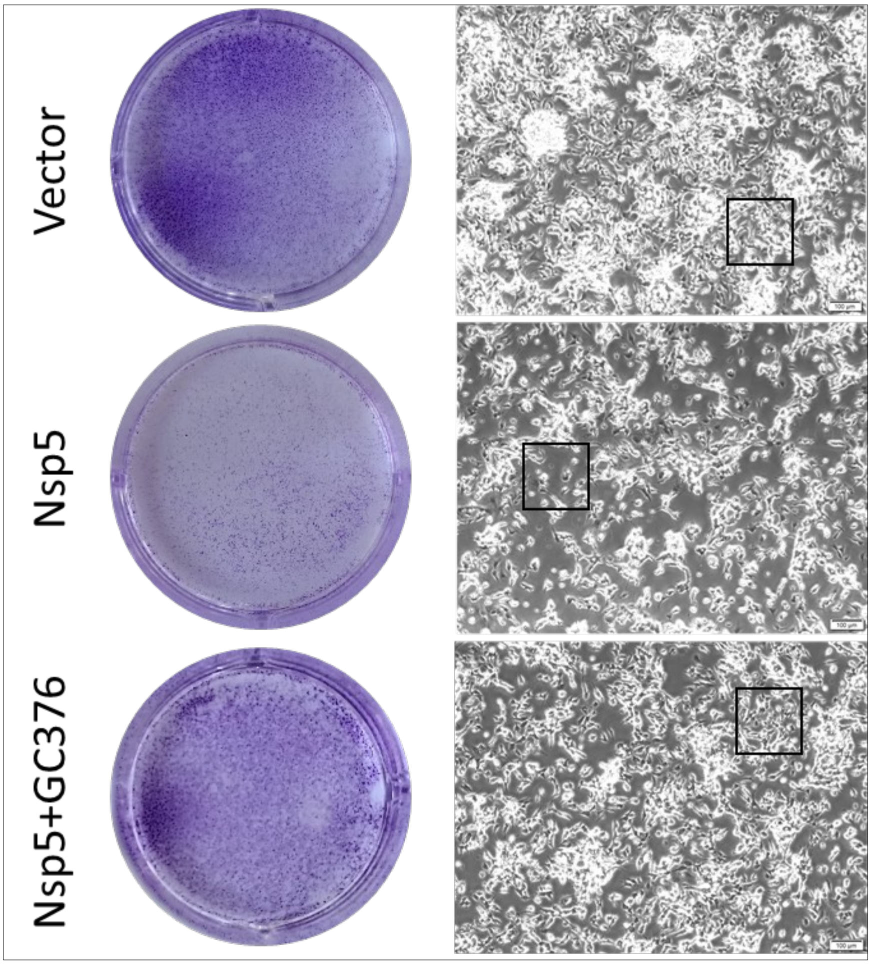

Figure S1b

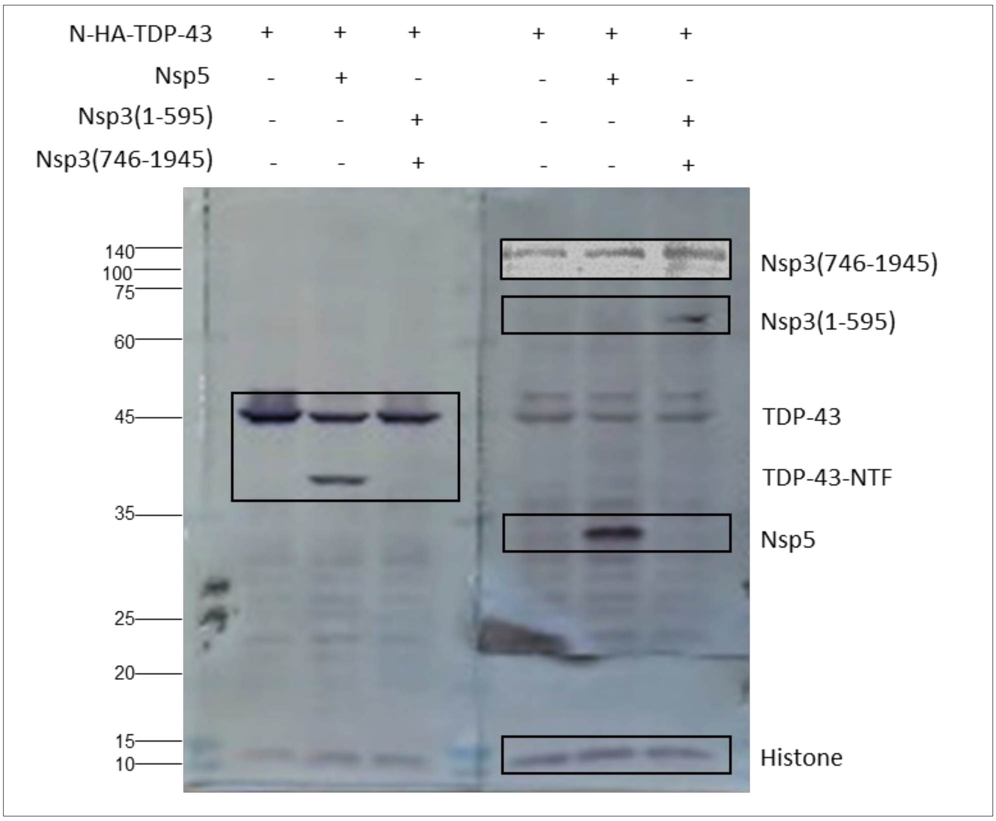
N-HA-TDP-43 + + + + + +
Nsp5 - + - - + -
Nsp3(1-595) - - + - - +
Nsp3(746-1945) - - + - - +
140
100
75
60
45
35
25
20
15
10
Nsp3(746-1945)
Nsp3(1-595)
TDP-43
TDP-43-NTF
Nsp5
Histone

Figure S1c

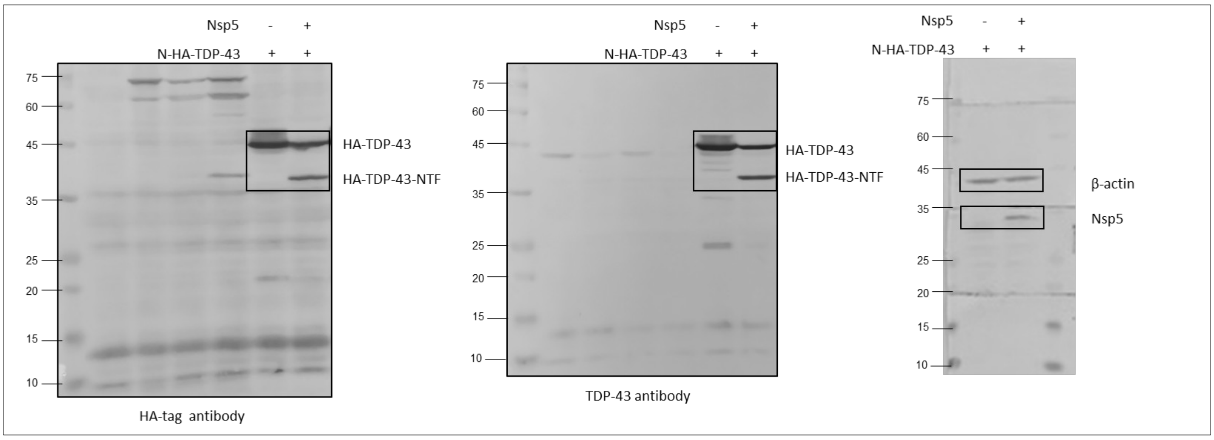
Nsp5 - +
N-HA-TDP-43 + +
75
60
45
35
25
20
15
10
HA-TDP-43
HA-TDP-43-NTF
HA-tag antibody
TDP-43 antibody
β-actin
Nsp5

Figure S2b

GC376(μM)	Blank	0	0.001	0.01	0.1	1	10	100
OD450	0.337	1.931	1.959	2.041	1.823	1.946	1.897	1.734
	0.291	1.743	1.884	1.915	1.661	1.691	1.561	1.401
	0.309	1.725	1.876	1.676	1.851	1.614	1.675	1.299

Figure S4

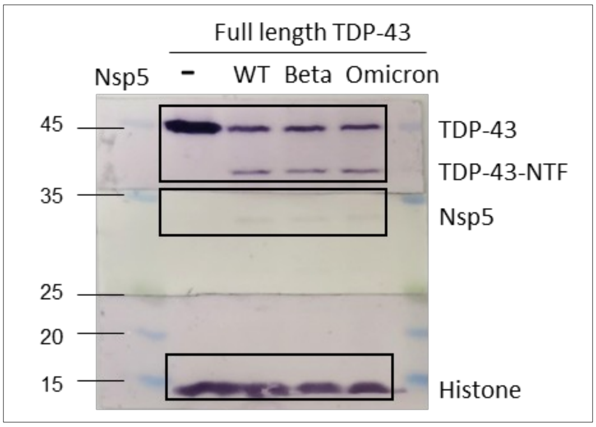

Full length TDP-43
Nsp5
-
WT
Beta
Omicron
45
35
25
20
15
TDP-43
TDP-43-NTF
Nsp5
Histone

Figure S5

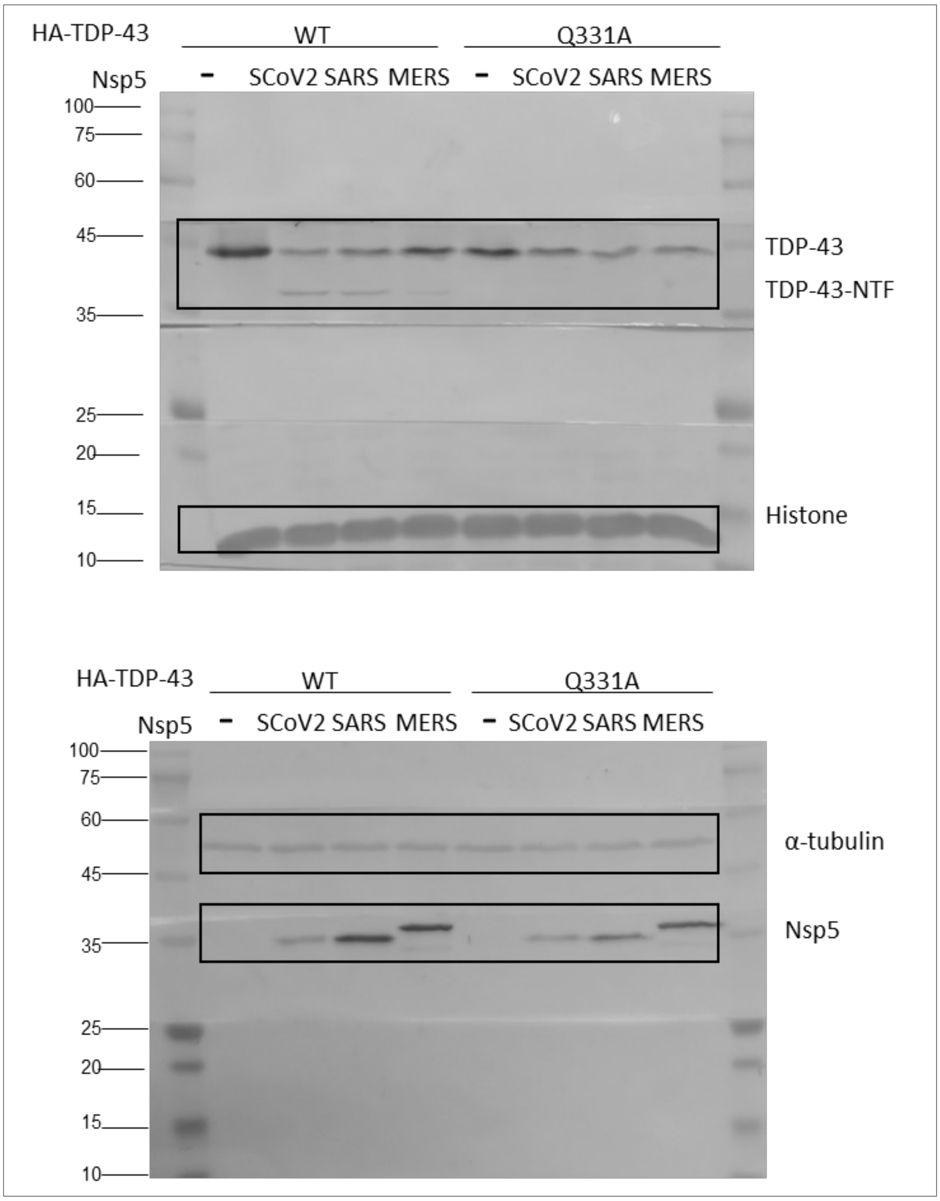
HA-TDP-43
WT
Q331A
Nsp5
-
SCoV2 SARS MERS
-
SCoV2 SARS MERS
100
75
60
45
35
25
20
15
10
TDP-43
TDP-43-NTF
Histone
HA-TDP-43
WT
Q331A
Nsp5
-
SCoV2 SARS MERS
-
SCoV2 SARS MERS
100
75
60
45
35
25
20
15
10
α-tubulin
Nsp5

Figure S6

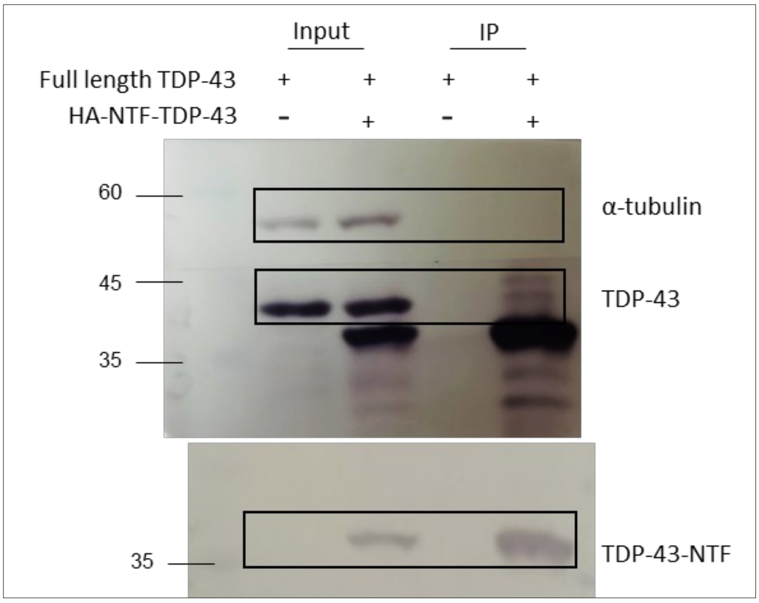

Input
IP
Full length TDP-43 + + + +
HA-NTF-TDP-43 - + - +
60
45
35
α-tubulin
TDP-43
35
TDP-43-NTF

Figure S7

T98G

TDP-43	-	WT			Q331A		
Nsp5(ng)	0	0	100	200	0	100	200
	0.391	0.408	0.424	0.426	0.413	0.433	0.457
	0.375	0.386	0.415	0.428	0.428	0.437	0.47
	0.371	0.381	0.4	0.42	0.419	0.421	0.457

Figure S8

SH-SY5Y	Vector	TDP-43-Q331A	Nsp5	Nsp5+TDP-43-Q331A
	0.408	0.416	0.431	0.408
	0.413	0.421	0.442	0.421
	0.404	0.422	0.442	0.421

Figure S9

SH-SY5Y

GC376(μM	Blank	0	0.01	0.1	1	10	100
OD450	0.408	0.557	0.566	0.573	0.547	0.578	0.556
	0.415	0.563	0.575	0.564	0.594	0.547	0.595
	0.39	0.556	0.576	0.545	0.556	0.571	0.565
